# Lightweight Visual Dynamic Gesture Recognition System Based on CNN-LSTM-DSA

**DOI:** 10.3390/s26051558

**Published:** 2026-03-02

**Authors:** Zhenxing Wang, Ziyan Wu, Ruidi Qi, Xuan Dou

**Affiliations:** 1 School of Computer and Information Engineering, Shanghai Polytechnic University, Shanghai 201209, China; wuziyan170@163.com (Z.W.); 19506151605@163.com (R.Q.); 2 School of Intelligent Manufacturing and Control Engineering, Shanghai Polytechnic University, Shanghai 201209, China; 20241513122@sspu.edu.cn

**Keywords:** visual gesture recognition, CNN-LSTM hybrid model, depthwise separable convolution, bionic robotic hand

## Abstract

**Highlights:**

**What are the main findings?**

- A lightweight CNN-LSTM-DSA hybrid model was developed for visual dynamic gesture recognition, achieving high-precision recognition of both static (96%) and dynamic (90.19%) gestures with low response latency (<300 ms).
- The system demonstrated excellent robustness under varying lighting and background conditions, with successful real-time mapping of gestures to robotic hand movements.

**What are the implications of the main findings?**

- This model provides an efficient solution for embedded gesture recognition, ensuring high accuracy while minimizing computational overhead, making it suitable for deployment on resource-constrained devices like the Jetson Nano.
- The proposed approach enhances human–robot interaction, offering practical applications in virtual reality, intelligent robotics, and other real-time interactive systems.

**Abstract:**

Addressing the challenges of large-scale gesture recognition models, high computational complexity, and inefficient deployment on embedded devices, this study designs and implements a visual dynamic gesture recognition system based on a lightweight CNN-LSTM-DSA model. The system captures user hand images via a camera, extracts 21 keypoint 3D coordinates using MediaPipe, and employs a lightweight hybrid model to perform spatial and temporal feature modeling on keypoint sequences, achieving high-precision recognition of complex dynamic gestures. In static gesture recognition, the system determines the gesture state through joint angle calculation and a sliding window smoothing algorithm, ensuring smooth mapping of the servo motor angles and stability of the robotic hand’s movements. In dynamic gesture recognition, the system models the key point time series based on the CNN-LSTM-DSA hybrid model, enabling accurate classification and reproduction of gesture actions. Experimental results show that the proposed system demonstrates good robustness under various lighting and background conditions, with a static gesture recognition accuracy of up to 96%, dynamic gesture recognition accuracy of 90.19%, and an overall response delay of less than 300 ms.

## 1. Introduction

With the rapid development of artificial intelligence technology, gesture recognition, as a natural human–computer interaction method, has been widely applied in fields such as virtual reality and intelligent robots [1]. Gesture recognition not only provides a more intuitive and natural way of interaction but also enhances the efficiency of human–computer collaboration. However, existing gesture recognition systems still face challenges such as high computational complexity and large response latency in practical applications, especially when deployed on embedded devices where hardware limitations make efficient real-time recognition difficult [2].

In recent years, with the rapid development of deep learning, significant progress has been made in gesture recognition technology [3]. Hand keypoint detection serves as a critical front-end for landmark-based gesture recognition. Although various approaches have been explored, embedded deployment often favors lightweight on-device solutions to satisfy strict latency and resource constraints. Traditional gesture recognition methods usually rely on temporal modeling and feature extraction techniques [4]. Gil-Martín et al. proposed a landmark-based dynamic gesture recognition framework that combines MediaPipe coordinate extraction with deep learning networks; by using landmark coordinates instead of full-frame images, the approach reduces the information conveyed to the recognition module and achieves a more parameter-efficient design on the IPN Hand dataset (50 subjects, 13 gesture classes) [5]. Tran et al. proposed a 3D-CNN model for video feature learning; while improving spatiotemporal representation, such often involve higher computational cost and increased training demands [6]. Baumgartl et al. fine-tuned a MobileNetV2-based CNN for static hand gesture recognition on the Pugeault and Bowden image dataset (over 48,000 RGB images; 60,000 images were extracted and balanced to 2500 samples per class), covering 24 ASL alphabet gestures, and achieved a balanced accuracy of 99.96% with a compact model size of ~24 MB [7]. However, these methods often rely on complex computational frameworks and large datasets when dealing with dynamic gestures, resulting in high computational resource requirements and complex inference processes. To improve recognition accuracy, many studies have started exploring hybrid deep learning architectures based on convolutional neural networks. These methods often involve the processing of multimodal data, such as optical flow, depth maps, and segmentation maps, which helps improve the accuracy of dynamic gesture recognition [8,9]. The multi-scale CNN-BiLSTM architecture proposed by Zhang et al. combined with a hierarchical attention mechanism showed excellent robustness in handling high noise, missing data, and outliers [10]. Moreover, training with the Transformer model can further enhance performance, especially in handling long sequences and complex temporal dependencies [11]. In parallel, multi-sensor systems (e.g., radar and time-of-flight sensors) have been studied to enhance robustness under challenging visual conditions [12], and hardware-aware optimization strategies such as neural architecture search have been used to co-optimize accuracy and latency on low-power edge devices [13]. These lines of research indicate active progress across diverse settings; however, differences in sensing modalities, supervision targets, and evaluation protocols may limit direct comparability with a vision-only, on-device recognition pipeline.

To ensure a fair and reproducible comparison, the experimental evaluation in this work focuses on vision-based methods under the same task definition and supervision protocol. We benchmark the proposed model against representative spatiotemporal baselines commonly used for video/sequence gesture recognition, including a 3D-CNN–LSTM architecture for spatiotemporal feature learning [14], a lightweight MobileNetV2–LSTM variant [15], an attention-based CNN–BiLSTM model for temporal aggregation [16], and a CNN-based detector combined with a deep belief network classifier [17]. In addition, we report a MediaPipe-only baseline to isolate the contribution of the recognition model beyond landmark extraction [18]. Methods involving additional sensing modalities or substantially different supervision targets are discussed for context but are not used as direct baselines under our experimental setting. Although Long Short-Term Memory (LSTM) networks are highly effective in temporal modeling, research focused on optimizing deep models specifically for edge devices with stringent real-time inference requirements, including tight constraints on memory usage and end-to-end latency, remains limited [19]. Therefore, this study proposes a visual dynamic gesture recognition system based on a CNN–LSTM–DSA hybrid model, designed for on-device deployment on an NVIDIA Jetson Nano (a low-power embedded GPU platform). Given that both computational and memory resources are constrained compared to desktop GPUs, and real-time responsiveness is critical for interactive applications, the system is optimized to operate effectively within these limitations. In this context, “resource-constrained” refers to the requirement for running the complete pipeline directly on the device without offloading tasks to the cloud, while maintaining a compact model size and low-latency inference.

The primary objective of this study is to achieve sub-300 ms end-to-end response latency, coupled with competitive recognition accuracy, suitable for real-time interactive applications in scenarios such as human–robot interaction, assistive technology, and gesture-based control systems. Specifically, the system uses MediaPipe for hand keypoint preprocessing [20], integrates depthwise separable convolution and dual-attention mechanisms [21,22], and combines them with a CNN–LSTM backbone to optimize inference efficiency on the Jetson Nano platform. Experimental results demonstrate a maximum static gesture accuracy of 96% across different environments and a dynamic gesture accuracy of 90.19%, with response latency consistently maintained below 300 ms, proving the system’s practicality for real-world deployment in edge computing and interactive systems. This system can be applied to typical task scenarios such as human–robot collaboration, robot manipulation, and assistive robotics. When deployed in robotic systems, it enables contactless operation and gesture-based interaction in industrial and civilian sectors, including smart factories and healthcare, demonstrating significant potential for real-world applications. To clarify the methodological choice for dynamic gestures, we formulate dynamic gesture recognition as command recognition for human–computer and human–robot interaction. Predicted gesture categories are mapped to discrete commands that trigger control signals and drive predefined robotic-hand actions. This formulation supports command-level interaction by providing stable semantic outputs under variations in gesture speed and keypoint noise, and it is consistent with the supervision protocol adopted in this study. By contrast, continuous motion reproduction without explicit classification aims to estimate continuous control variables and typically requires dense supervision and additional stabilization under keypoint jitter and occlusions. Accordingly, we adopt classification for reliable command triggering and leave continuous reproduction as future work.

## 2. System Architecture Overview

**Figure 1:**
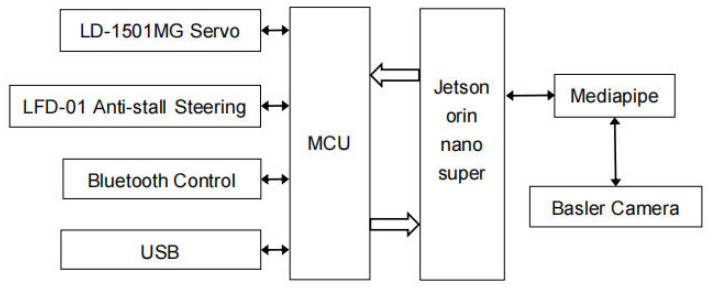
System hardware architecture.

The system’s hardware architecture, as shown in Figure 1, primarily consists of three components: the perception layer, processing layer, and execution layer. At the perception layer, the system employs a Basler industrial camera (Basler AG, Ahrensburg, Germany) to capture gesture video streams, utilizing the MediaPipe framework for hand keypoint detection and feature extraction. The processing layer consists of the NVIDIA Jetson Orin Nano Super (NVIDIA Corporation, Santa Clara, CA, USA) embedded computing platform, a core computing unit that integrates a high-performance GPU and AI acceleration units. This platform enables efficient execution of deep learning inference tasks on edge devices. At the execution layer, the system interacts with peripherals through the MCU control unit, including the LD-1501MG servo and LFD-01 anti-shake servo, to achieve flexible control and motion output of the robotic arm. Through multiple communication methods including serial ports, Bluetooth, and USB, the modules work together to build a complete closed-loop control system that spans gesture acquisition, feature recognition, decision-making and reasoning, to action execution.

## 3. Static Gesture Recognition and Real-Time Mapping to the Robotic Hand

### 3.1. MediaPipe Hand Detection

Vision-based hand keypoint detection has evolved rapidly in recent years. Existing methods can be broadly grouped into top-down pipelines that first localize the target region and then estimate landmarks, and bottom-up pipelines that jointly predict keypoints and their associations in the full image [23]. Recent pose-estimation advances further improve keypoint localization through strong backbone representations and heatmap-based regression models [24]. For hands, dedicated landmark estimators have been developed to better handle fine-grained articulation and occlusions while maintaining real-time performance on resource-limited platforms [25]. In parallel, progress in monocular RGB-based 3D hand pose estimation has been accelerated by improved learning paradigms and richer training supervision [26,27]. Despite strong accuracy, many general-purpose keypoint estimation frameworks remain computationally demanding for embedded deployment.

To satisfy these constraints, we adopt an on-device hand landmark extraction module as the front end of our pipeline. In particular, we build the landmark extraction stage on MediaPipe, a framework for constructing real-time perception pipelines with efficient processing of streaming visual data on-device [28]. Similar to Google’s open-source framework, MediaPipe analyzes images through hand feature recognition models to identify key hand features such as fingertips and knuckles. The model extracts the coordinates of these features, converting the spatial layout of the hand into a numerical representation. Using these 2D and 3D coordinates, it provides structured output conveying hand posture or gestures. This approach involves managing image data, extracting landmark points, and accurately interpreting hand movements. The framework can recognize 21 key hand points through training data, demonstrating high accuracy and robustness.

Additionally, MediaPipe provides stable and reliable technical support for real-time and low-latency gesture recognition, making it suitable for applications such as dynamic hand tracking. Building on this lightweight front end, our recognition model employs depthwise separable convolutions and a dual-attention mechanism to process landmark-based gesture inputs with minimal computational overhead, enabling efficient deployment on the Jetson Orin Nano Super. In our experiments, MediaPipe achieved a frame rate of 30 FPS with end-to-end latency for dynamic gesture recognition under 300 ms, demonstrating its effectiveness in real-time applications. Furthermore, MediaPipe supports real-time sign language recognition on both mobile and PC platforms, ensuring high-speed response even in live video stream scenarios. By training with MediaPipe features, it can recognize gestures even within live streams [29].

Vision-based technology enables seamless human–machine interaction through webcams, offering a cost-effective solution. Using the MediaPipe library for image processing, our method effectively detects and isolates hands in camera images, overcoming challenges posed by diverse hand appearances due to motion, skin tone, viewing angle, proportions, and camera settings. MediaPipe processes these 2D images to extract hand keypoints, and through its deep learning model, it estimates the 3D coordinates of these keypoints, even though the input is from a 2D camera. This ensures robust hand detection and accurate estimation of hand gestures despite inherent complexity.

### 3.2. Keypoint Data Processing

In static gesture recognition, this system classifies gestures by calculating the angles between finger joints: MCP, PIP, DIP, and TIP. To enhance recognition robustness and stability, a sliding window smoothing technique is employed to perform de-jittering processing on finger states across consecutive frames.

(1) Coordinate Normalization

Normalization is a critical step in gesture data preprocessing, essential for preparing this diverse dataset for analysis. It involves techniques for standardizing collected data, accounting for variations such as hand shape, size, and motion. By capturing natural hand postures, the collected data aims to comprehensively reflect real-world scenarios, enhancing the system’s adaptability to diverse gestures and movements. To eliminate positional variations during feature extraction, data must be normalized to scale within the [0, 1] range. This helps balance feature influence, streamline processing, and enhance training efficiency.

In this study, we determine the bending state of the fingers by calculating the angles between the finger joints. Specifically, the bending angles of the index, middle, ring, and little fingers are determined by calculating the angles between the MCP, PIP, DIP, and TIP joints, using the vector angle formula for each joint. For the thumb, the bending angle is calculated using the angles between the CMC-MCP-IP and TIP-IP-MCP joints. To assess the bending degree of the fingers, we set different angle thresholds: for the index, middle, ring, and little fingers, the angle range is set from 70° to 180°, where smaller angles indicate more bending, and larger angles indicate more straightening. For the thumb, the bending angle range is set from 90° to 180°, as the thumb typically has a different range of motion compared to the other fingers. Each angle value is then normalized to a range of 0 to 1. In gesture recognition, coordinate normalization is a critical step in data preprocessing, essential for eliminating the effects of hand position, size, and posture variations on the model. We use MediaPipe to extract hand keypoints, which provides 21 keypoints in 3D coordinates. To eliminate these variations, we normalize the coordinates of each keypoint relative to the wrist.

Specifically, using the wrist (index 0) as the reference point, the relative coordinates of each keypoint are computed and normalized using the following formula:

(1) $$\begin{array}{l} normalized\_x=\frac{landmark.x-wrist.x}{image.width}\\ normalized\_y=\frac{landmark.y-wrist.y}{image.width} \end{array}$$

where wrist.x, wrist.y are the 3D coordinates of the wrist, and image.width, image.height, image.width, image.height are the image dimensions. This normalization scales the keypoint coordinates to the range of [0, 1], eliminating the influence of hand size, position, and posture variations on the subsequent angle calculation.

(2) Smooth Processing with Sliding Window

Sliding window is a technique that splits time-series or continuous frame data into fixed-size windows for processing. Within each window, majority voting is performed to obtain the final smoothed result for each frame. In each window, the state of each finger is voted on, and if the majority determines that a finger is bent (0) or straightened (1), that state is output. This helps avoid incorrect decisions caused by occasional errors or noise.

Suppose the gesture state vector for each frame in the time sequence is:

(2) $$S_{t}=[s_{t1},s_{t2},s_{t3},s_{t4},s_{t5}]$$

Here, $S_{t}$ ∈{0, 1} indicates the bending (0) or straightening (1) state of the i-th finger in the t-th frame.

Define the sliding window width as N. For each finger, perform majority voting on its state within the window:

(3) $${\hat{s}}_{ti}=\left\{\begin{array}{l} 1\sum_{j=t-N+1}^{t}s_{ji}\geq \frac{N}{2}\\ 0\;\;\;\mathrm{else} \end{array}\right.$$

This effectively filters out transient jitter errors, generating a smooth gesture state output.

**Figure 2:**
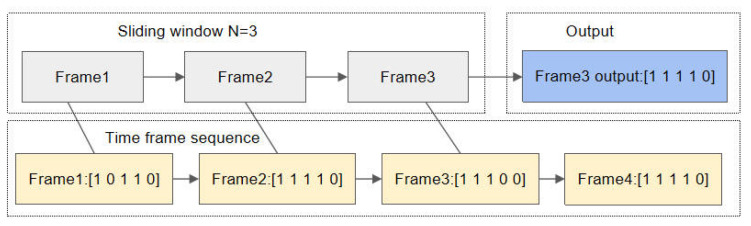
Sliding window time frame diagram.

In the gesture recognition process, we use a sliding window technique (window length N = 3) to smooth the recognition results of the previous frames, thereby reducing instantaneous fluctuations and improving recognition stability. Figure 2 shows the raw data of frame 1 and frame 4. At the moment of frame 3, the sliding window covers frames 1 to 3, and after smoothing these three frames, the smoothed result of frame 3 is output as [1 1 1 1 0].

(3) Angle Calculation

The bending angles of the index, middle, ring, and little fingers are determined by calculating the angles between the joints. Specifically, we calculate the angles between the MCP, PIP, DIP, and TIP joints. For each finger, we compute the vectors between consecutive joints and calculate the angles between them using the vector angle formula.

For the index finger, for example, we calculate the angles between the following vectors:

Vector 1: From MCP to PIP;

Vector 2: From PIP to DIP;

Vector 3: From DIP to TIP.

The angle *θ* is then calculated using:

(4) $$\theta =\cos^{-1}(\frac{\vec{A}\cdot \vec{B}}{\left|\vec{A}\right|\cdot \left|\vec{B}\right|})$$

$\vec{A}$ and $\vec{B}$ are the vectors between joint points.

For the index, middle, ring, and little fingers, we calculate the angles using MCP-PIP-DIP and TIP-DIP-MIP. The total bending angle for each finger is the average of the two calculated angles:

(5) $$Angle=(\theta_{1}+\theta_{2})/2$$

For the thumb, we calculate the angle using CMC-MCP-IP and TIP-IP-MCP.

(4) Speed Regulation and Servo Smooth Control

Once the bending angles of the fingers are calculated, they are normalized to a range of [0, 1]. For instance, the bending angle for each finger typically ranges from 70° to 180°, and the normalized angle is calculated using the formula:

(6) $$normalizedAngle=\frac{\theta -70}{180-70}$$

Here, θ represents the bending angle of the finger. The normalized angle is then mapped to the PWM control signal for the servos, which ranges from 900 to 2000 microseconds, corresponding to finger bending and straightening, respectively:

(7) $$P_{PWM}=900+normalizedAngle\cdot (2000-900)$$

To prevent abrupt motion of the robotic finger, the control commands are sent with an execution time parameter, ensuring smooth transitions between the bending and straightening states. This motion smoothing is achieved by adjusting the duration parameter in the servo drive code, which is implemented by calling a function. This process guarantees that the robotic finger moves fluidly without jerks, ensuring natural and precise motion in real-time interactions.

### 3.3. Real-Time Mapping to the Robotic Hand

First, the system detects the hand in the camera, processes the detected hand information through MediaPipe, and displays it as 21 hand keypoints. After processing with an algorithm, the 3D hand information is visualized, with each keypoint showing (x, y, z) coordinates. Then, based on the coordinates and formulas of the keypoints, the angle and distance between each keypoint are calculated to determine the current state of the hand. The bending angles of the index finger, middle finger, ring finger, and little finger are determined by the MCP-PIP-DIP and TIP-DIP-MIP angle information. The angle threshold range for the thumb is determined based on the angle information between CMC-MCP-IP and TIP-IP-MCP, spanning 70–180 degrees. Once the angles are calculated, they are normalized to a 0–1 scale, and this normalized data is then linearly mapped to the joint angles of the robotic hand’s servos. The servo motors for each finger are controlled with a range of 900 to 2000 degrees, corresponding to finger bending and straightening states. The data is then protocol-packaged and transmitted to the MCU for processing. The MCU decodes the control commands and sends the appropriate signals to each servo motor in the robotic hand, allowing real-time movement based on the recognized hand gestures. This implementation ensures that the robotic hand follows the movements of the human hand in real time with high precision, enabling smooth and natural interaction for applications such as human–robot collaboration and gesture-based control systems.

## 4. Dynamic Gesture Recognition Based on CNN-LSTM-DSA

Dynamic gestures are formulated as discrete command recognition from hand keypoint sequences. The predicted gesture category is mapped to predefined commands to trigger control signals for robotic-hand execution.

### 4.1. Dataset

The Jester-20BN dataset is a large-scale real-world video dataset for gesture recognition [30], comprising 148,092 video clips depicting 27 distinct actions. Each clip lasts approximately 3 s. To ensure the training model achieves high accuracy, we conducted an initial screening of the data, selecting 15 categories as samples for the training model. The final training dataset comprised 50,420 video segments. When processing the Jester dataset, we first preprocess the videos to extract hand keypoints from each frame. To enhance the model’s generalization capabilities, we apply data augmentation techniques including rotation, scaling, cropping, and flipping. These operations effectively increase the diversity of training data, enabling the model to better handle challenges posed by varying lighting conditions, backgrounds, and gesture variations.

### 4.2. Feature Extraction

MediaPipe incorporates a built-in hand keypoint model that can return finger joint locations in images with low computational overhead. Leveraging this capability, we locate the hand region within an image and crop that area. Hand keypoint recognition can be performed on RGB images, which helps avoid issues where parts of the joints are unclear due to hand occlusion. Based on this, we first use MediaPipe to extract hand keypoints from the dataset, and the processed 2D data is then used for model training.

### 4.3. CNN-LSTM-DSA Hybrid Model

**Figure 3:**
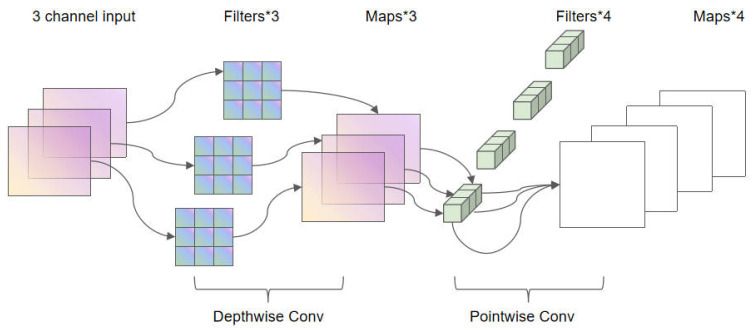
The figure of the deep separable convolutional model.

The traditional CNN-LSTM model directly inputs image frames. Given limited computational resources, we input the data as coordinates extracted from hand keypoints. To significantly reduce the number of parameters and computational complexity while maintaining model performance, we used depthwise separable convolution to replace standard convolution operations, as shown in the model diagram in Figure 3.

Depthwise separable convolution decomposes the standard convolution into two consecutive operations: depthwise convolution and pointwise convolution.

Depthwise convolution performs spatial convolution independently on each input channel, with the number of parameters being $K\times K\times C_{in}$, where K is the size of the convolution kernel and $C_{in}$ is the number of input channels. The subsequent pointwise convolution uses a 1 × 1 convolution kernel to map the output of the depthwise convolution to the target number of output channels, $C_{out}$, with the number of parameters being $C_{in}\times C_{out}$.

Compared to the parameter count of $K\times K\times C_{in}\times C_{out}$ in standard convolution, the total parameter count of depthwise separable convolution is $K\times K\times C_{in}+C_{in}\times C_{out}$, with a parameter reduction rate of:

(8) $$R=1-\left(\frac{1}{C_{out}}+\frac{1}{K^{2}}\right)$$

For a typical configuration with a 3 × 3 convolution kernel and 32 output channels, the number of parameters can be reduced by approximately 85.7%. This concept of depthwise separable convolution was introduced in MobileNet and has been found effective in computer vision tasks. Additionally, depthwise separable convolution has also performed exceptionally well in tasks such as video prediction, motion object detection, and object segmentation.

**Figure 4:**
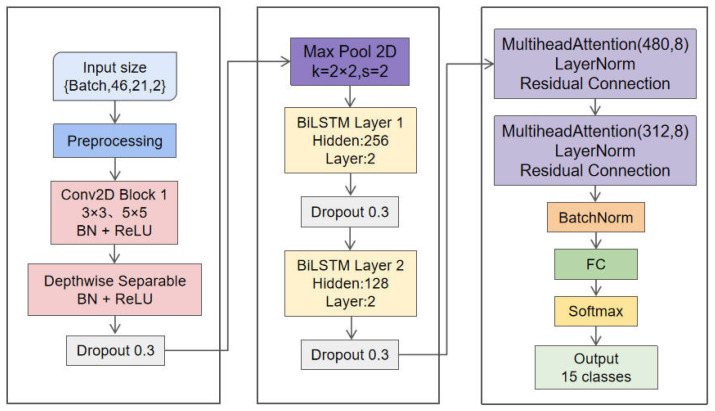
Architecture Diagram of the CNN-LSTM-DSA Hybrid Model.

Combining them enhances the model’s ability to understand and process complex inputs, especially in the context of gesture recognition. The CNN component initially extracts features, focusing on the spatial information in the input data. With its ability to analyze image data, the CNN inspects the input, identifies patterns and relevant features, and passes the output directly to the LSTM layer. The LSTM is renowned for its excellence in sequence learning and subsequently processes information from the CNN. The CNN extracts spatial features, capturing the spatial details of gestures, while the LSTM handles sequence patterns and addresses the temporal dimension of the data. By integrating these two architectures, the hybrid model can comprehensively understand the spatial complexity and temporal patterns in hand movements. CNN extracts the spatial relationship features of hand keypoints from each frame, and then LSTM learns the variation patterns of these features in the time dimension, enabling accurate recognition of dynamic gestures. The overall architecture of the model is shown in Figure 4.

The shape of the input layer is (B, 46, 21, 2), representing 46 frames of gesture keypoints, with 2 coordinates for each keypoint. During the feature extraction phase, depthwise separable convolution layers are first used to augment the input features. Each convolution layer uses 1 × 1 and 3 × 3 convolution kernels, combined with batch normalization and ReLU activation functions, which enhance the model’s non-linear expression capability and accelerate the training process. Next, through the multi-scale feature extraction module, convolution kernels of different sizes (3 × 3 and 5 × 5) are used to capture spatial information at different scales. On this basis, the features are downsampled using a max-pooling layer to reduce the spatial dimension, extracting more compact and effective feature representations. After feature fusion, we obtain high-dimensional feature representations, providing sufficient spatial information for subsequent temporal modeling.

In the temporal modeling phase, a dual-layer LSTM structure is used to model the temporal features of dynamic gestures. The first LSTM layer has 256 hidden units, and the second LSTM layer has 128 hidden units, aiming to capture the temporal dependencies of gestures. To reduce overfitting and enhance the model’s generalization capability, a Dropout layer was applied after the LSTM layer. Batch normalization was performed on the output of each LSTM layer to accelerate network training and improve stability.

In the dual attention mechanism, to better handle the temporal and spatial information in dynamic gestures, two important attention layers are combined. Firstly, the first attention layer weights the spatial features with an input dimension of 480 and uses 8 attention heads. The output of each attention head is normalized and directly passes the signal through a residual connection to avoid the potential problem of gradient vanishing during training. Then, the input dimension of the second attention layer comes from the output of the second LSTM layer, with a dimension of 312, and also uses 8 attention heads, mainly for capturing key temporal features from the time series. Similar to the first layer, the output is normalized and avoids information loss through a residual connection, enhancing the model’s learning ability. Finally, classification is performed through a fully connected layer, with the number of output categories consistent with the number of categories in the dataset.

The design goals of the entire model are: (A) to use spatial–temporal features as input; (B) to achieve high classification accuracy; (C) to maintain low computational complexity; and (D) to ensure efficient deployment on embedded platforms. By means of this hybrid model, which combines the advantages of CNN in spatial feature extraction and the strengths of LSTM in temporal modeling, efficient recognition of dynamic gestures is achieved, while ensuring the model’s lightweight and efficient inference capabilities.

## 5. Model Training and Experimental Results

### 5.1. Model Training

For all datasets, data splitting involves allocating 80% of the total gesture fragments for each class to training and 20% to validation. This enables the model to learn from a substantial portion of the data, facilitating generalization assessment and preventing overfitting.

This section describes the training setup and implementation details of the proposed system. Experiments were conducted on a laptop equipped with an Intel Core i7-14650HX processor and an NVIDIA GeForce RTX 5060 GPU. The architecture design and training were performed using the PyTorch 2.7.1 deep learning framework on the Windows operating system. The system performs inference and recognition on captured dynamic gesture data using a CNN-LSTM-DSA hybrid model. This approach extracts spatial and temporal features from hand keypoint time series to achieve dynamic gesture classification and recognition.

In addition to the high-end system, experiments were also conducted on the Jetson Orin Nano Super, an embedded platform designed for edge computing with enhanced computational power. Equipped with an 8-core ARM Cortex-A78 CPU, 1024-core NVIDIA Ampere GPU, and 16 GB LPDDR5 RAM, the Jetson Orin Nano Super provides significant advantages for real-time inference in dynamic gesture recognition tasks. With a performance of 41 TOPS (Tera Operations Per Second) and typical power consumption of 15 W, this platform is well-suited for applications requiring low power consumption and high computational efficiency. The inference time and latency for this platform were measured to evaluate its suitability for edge computing tasks, ensuring real-time performance for gesture recognition.

**Figure 5:**
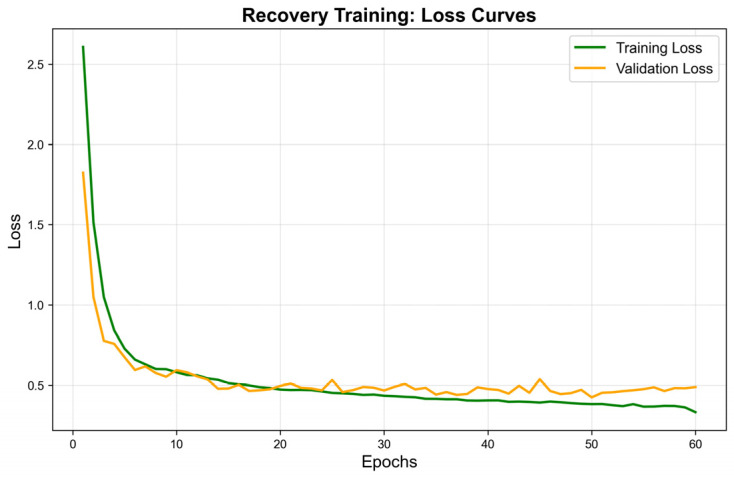
Loss Curve of the Best CNN-LSTM-DSA Model.

For the Jester dataset, we first employ MediaPipe for landmark-based preprocessing and establish an initial landmark-based baseline. We then evaluate a CNN–BiLSTM baseline on the same landmark inputs. Building on these observations, we introduce depthwise separable convolutions and a dual-attention mechanism into the proposed CNN–LSTM backbone and tune key hyperparameters (160 hidden units, class weighting, learning rate of 0.004, and 60 epochs). The optimized model achieves 90.19% accuracy on Jester. The training loss curve in Figure 5 indicates stable convergence.

### 5.2. Experimental Results

The comparison methods are selected based on three considerations: (i) they are representative spatiotemporal architectures widely used for dynamic gesture/video recognition; (ii) they are feasible to reproduce and evaluate under our setting; and (iii) they cover both video-based and landmark-based pipelines to reflect typical design choices for real-time human–computer/robot interaction on resource-constrained platforms. In particular, we include both a MediaPipe-only baseline to quantify the contribution of landmark extraction alone and lightweight/temporal models to evaluate different sequence modeling strategies.

To ensure fair comparison, all methods are evaluated under the same dataset protocol, identical input resolution and sequence length, and a unified training procedure (optimizer, batch size, and number of epochs when applicable). We compare the proposed approach with representative baselines: (1) 3D-CNN–LSTM, which extracts spatiotemporal features using 3D convolutions and models temporal dependencies with an LSTM; (2) MediaPipe-only baseline, which uses the MediaPipe landmark representation with a lightweight classifier to quantify the contribution of landmark extraction alone; (3) CNN–BiLSTM, which applies a CNN feature extractor and bidirectional LSTM for temporal modeling; (4) CNN–DBW, which couples a CNN-based feature extractor/detector with a deep belief network classifier; and (5) a MobileNetV2–LSTM, which uses a lightweight CNN backbone followed by an LSTM for sequence aggregation

Comparing Table 1 and Table 2 reveals that the model trained on the MediaPipe-processed dataset demonstrates significantly higher accuracy during testing than other methods. Furthermore, incorporating deep separable convolutions and dual attention mechanisms not only enhances performance but also effectively reduces model size, latency, and energy consumption.

After MediaPipe data processing, the baseline model without depthwise separable convolution (DSA) and dual attention mechanisms in the CNN-LSTM model achieved an accuracy of 89.25%. On this basis, after adding depthwise separable convolution and dual attention mechanisms, the model’s accuracy remained almost unchanged, while other performance metrics were significantly improved, as shown in Table 2. By comparing the hardware metrics of the baseline model and the improved model on the same dataset, including the comparison of average accuracy (mAcc), computation time (FLOPs), inference time, model size, and model parameters on the test set, it is clear that our model successfully achieved real-time performance and lightweight optimization while maintaining high accuracy, reducing model size, latency, and energy consumption.

By comparing Table 1 and Table 2, we can see that the model’s accuracy on the dataset processed by MediaPipe is significantly better than other methods during testing. Moreover, by adding depthwise separable convolution and dual attention mechanisms, the model’s size, latency, and energy consumption have been reduced while improving performance.

**Figure 6:**
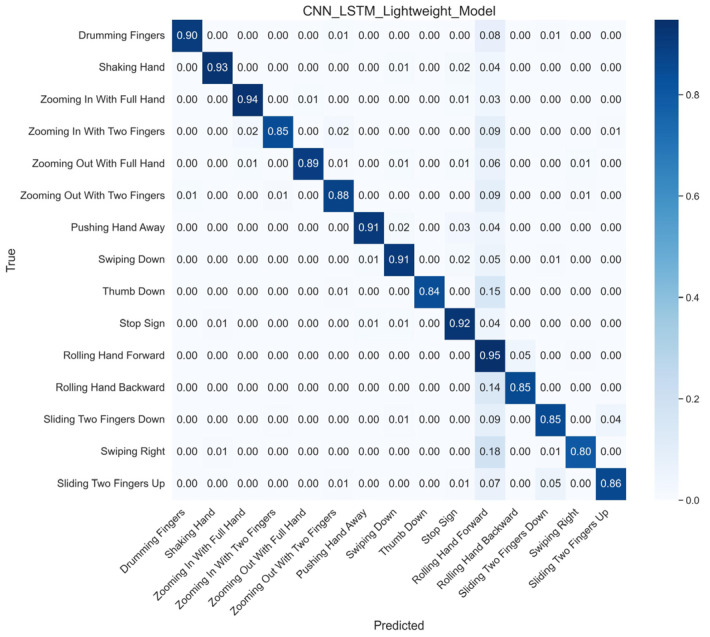
Confusion Matrix of the Best CNN-LSTM-DSA Model Results.

Additionally, Figure 6 shows the confusion matrix during the training process, indicating that the CNN-LSTM-DSA model achieved a classification accuracy of 90.19%, and the model demonstrated excellent accuracy and robustness in predicting multiple gesture categories.

In this study, experiments were conducted on two different hardware platforms to evaluate the system’s performance: a high-end system and an NVIDIA Jetson Nano embedded platform.

Table 3 compares the accuracy, computational complexity (FLOPs), inference time, latency, and power consumption for both platforms. The high-end system delivers faster inference and lower latency. However, the Jetson Nano, with its significantly lower power consumption (5 W), offers a compelling solution for edge computing applications. Although it exhibits slightly higher inference time and latency, the Jetson Nano maintains real-time performance with an accuracy of 89.13%. Its energy efficiency and ability to perform real-time processing make it an ideal choice for embedded systems where power constraints are critical, without compromising essential performance.

## 6. Experimental Results and Analysis

In this study, the Jetson Nano serves not only as the core computing unit but also supports the efficient operation of MediaPipe and CNN-LSTM-DSA model, while completing the control tasks of the robotic hand through the connection of external devices. The results presented in Table 3 indicate that while the high-end system excels in computational power, the Jetson Nano is capable of running the model with slightly higher inference time and latency, making it more suitable for edge devices where performance optimization and power efficiency are crucial. This demonstrates the model’s ability to be deployed in real-time environments with varying hardware capabilities, maintaining high accuracy while optimizing for size, latency, and energy consumption.

**Figure 7:**
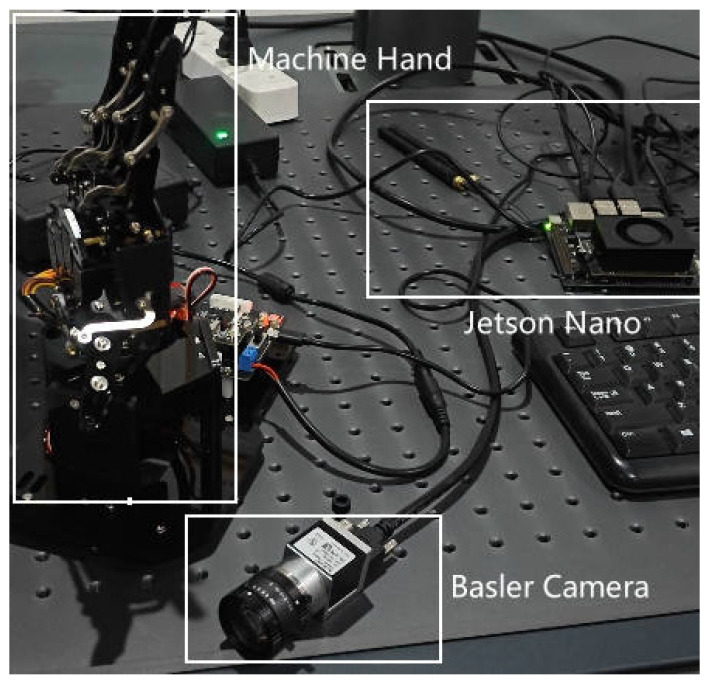
Gesture recognition system development environment.

To validate the practical effectiveness and stability of the vision-based gesture recognition control system, a complete experimental setup was constructed for system testing. The physical setup, shown in Figure 7, includes the Jetson Nano embedded platform, Basler industrial camera, software platform for running recognition and inference algorithms, and the STM32F103 control board (STMicroelectronics, Geneva, Switzerland) and robotic hand that receive control commands.

The system testing primarily focuses on static and dynamic gesture recognition, evaluating performance metrics such as recognition accuracy, response speed, and command transmission stability during the actual operation process. During testing, the input resolution and capture distance were kept within a similar range across conditions, while illumination and background complexity were varied according to the above definitions, in order to assess robustness under realistic environmental changes.

To improve clarity and reproducibility, the acquisition conditions are defined along two factors: illumination level and background complexity. Good lighting refers to an evenly lit indoor environment where the hand region is clearly visible with normal exposure and limited shadows. Low lighting refers to a dim environment in which frames exhibit reduced contrast and increased sensor noise, potentially weakening landmark visibility. Strong lighting refers to scenes with strong directional light or backlighting that introduces highlights, partial over-exposure, or pronounced shadows on the hand region. Simple background denotes a uniform or low-texture scene (e.g., plain wall/desk surface) with minimal distractors, whereas complex background denotes cluttered scenes containing rich textures or distractors (e.g., multiple objects, patterned surfaces, or human activities). During data collection, the camera viewpoint was kept approximately consistent, and the capture distance was maintained within 0.5–1.0 m across conditions, so that the main variations arise from illumination and background.

Based on the above definitions, six acquisition conditions were constructed by combining three illumination levels (good/low/strong) with two background types (simple/complex) to evaluate robustness in static gesture recognition. For each condition, at least 100 frames were collected for each gesture category. The evaluated static gestures include an open hand, a fist, and the “OK” sign. Recognition accuracy was computed as the percentage of correctly predicted frames, and stability was assessed by observing whether the predicted outputs remain consistent without frequent flipping under the majority-voting smoothing strategy.

The system uses MediaPipe to extract the coordinates of 21 hand keypoints, calculates the geometric angles, and combines a sliding window majority voting mechanism to achieve smooth output of gesture states, which are then mapped in real time to the servo angles of the robotic hand. The robotic hand’s movements are linked in real time to the recognition results, validating the closed-loop effect from recognition to control.

**Figure 8:**
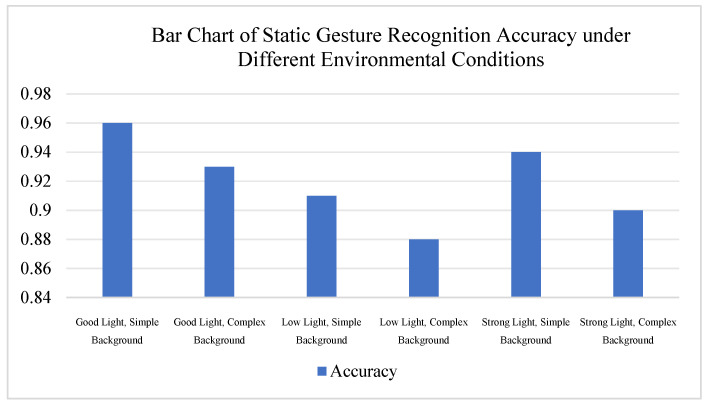
Bar Chart of Static Gesture Recognition Accuracy in Different Environments.

Figure 8 shows the bar chart of static gesture recognition accuracy under different environmental conditions. The experimental results show that under the condition of good lighting and a simple background, the recognition accuracy can reach 96%. Even under adverse environments such as low light and complex backgrounds, the system can still maintain an accuracy of around 88%. Throughout the tests, the system’s response delay remained stable between 200 and 300 ms, with smooth servo movements and no noticeable jitter. The experimental results show that the system has good robustness under different environments; however, variations in lighting and background still have some impact on the recognition performance. Future research could further introduce adaptive lighting correction and background denoising techniques to improve the system’s performance in extreme environments.

## 7. Conclusions

The visual dynamic gesture recognition system based on CNN-LSTM-DSA proposed in this paper successfully achieves high-precision gesture recognition with accurate mapping of both static and dynamic gestures, and real-time mapping of recognition results to the robotic hand’s motion control. Future work will focus on further optimizing the system’s response time and recognition accuracy, especially its stability under complex backgrounds and varying lighting conditions. Furthermore, combining the latest techniques in deep learning, such as Transformer and self-supervised learning, could further enhance the model’s performance and adaptability, enabling broader deployment in more practical application scenarios.

## Figures and Tables

**Table 1:** Comparison of Different Recognition Methods on the Jester Dataset.

Model	Recall	Acc
3DCNN-LSTM [14]	-	71%
Mediapipe [18]	78%	78%
CNN-BiLSTM [16]	82%	85.39%
MobileNet-V2-LSTM [15]	84.3%	84%
CNN-DBW [17]	88.21%	89.33%
Mediapipe-CNN-LSTM	88.9%	89.25%

**Table 2:** Comparison of Hard Metrics Between Models.

Model	mAcc (%)	FLOPs	Time (ms)	Model Size (MB)	Params
CNN-LSTM	89.25	193,536	120	6.08	4640
Our	90.19	27,552	81	2.85	656

**Table 3:** Performance Comparison Between High-end System and Jetson Nano.

Platform	mAcc (%)	FLOPs	Inference Time (ms)	Latency (ms)	Power Consumption
Intel Core i7 + RTX 5060	90.19	27,552	81	70	112 W
Jetson Nano	89.13	20,753	105	85	5 W

## Data Availability

The raw data supporting the conclusions of this article will be made available by the authors on request.
